# Ion Implantation Combined with Heat Treatment Enables Excellent Conductivity and Corrosion Resistance of Stainless Steel Bipolar Plate Anode for Hydrogen Fuel Cells

**DOI:** 10.3390/ma18071483

**Published:** 2025-03-26

**Authors:** Li Ding, Chaoqin Ren, Ruijuan Wang, Meng Yang, Yong Pan

**Affiliations:** 1 School of Automotive & Rail Transit, Nanjing Institute of Technology, Nanjing 211167, China; 2 College of Safety Science and Engineering, Nanjing Tech University, Nanjing 211816, China; rencq@njtech.edu.cn (C.R.); china202161201102@njtech.edu.cn (R.W.); 3 College of Materials Science and Engineering, Nanjing Tech University, Nanjing 211816, China; yangmengyy@njtech.edu.cn

**Keywords:** interfacial contact resistance, heat treatment, corrosion resistance, ion implantation

## Abstract

The broad use of (stainless steel) SS 316 L bipolar plates (BPs) in proton exchange membrane fuel cells relies (PEMFC) on high conductivity and corrosion resistance. To enhance the properties of stainless steel, this study applies ion implantation and heat treatment to form a non-homogeneous modified layer on SS 316 L. The injection of C and Mo ions on the SS 316 L surface caused irradiation damage, producing holes. But with the heat treatment of the ion-implanted samples, the irradiation-damaged surface will be repaired to a certain extent. The corrosion current density (I_corr_) of the 600 °C sample in the kinetic potential test (5.32 × 10^−4^ A/cm^2^) was 54% lower than that of the naked SS 316 L (1.17 × 10^−3^ A/cm^2^). In the electrostatic potential test, the corrosion current of the 600 °C sample stabilized at a low value (about 0.26 μA/cm^2^), with the lowest concentration of dissolved metal ions (Fe^2+^ 2.908 mg/L). After anodic electrostatic potential polarization, the interfacial contact resistance (ICR) of (Mo+C)_600-1_ was much lower than that of the untreated SS 316 L. Heat treatment experiments show that samples treated at 600 °C for 1 h exhibit significantly higher conductivity and anodic corrosion resistance than naked SS 316 L. This improvement is mainly due to the heat treatment under these conditions, which facilitated the formation of Mo carbides from the implanted C and Mo elements. Ion implantation and heat treatment enhance stainless steel surface conductivity and passive film corrosion resistance. These findings are useful in altering stainless steel BPs.

## 1. Introduction

With depleting non-renewable energy and worsening pollution, fuel cells are set to play a key role in the near future. The fundamental components of a PEMFC include BPs, which provide support and facilitate electron transfer from the gas, and membrane electrode assemblies (MEAs), which carry out redox reactions. In a PEMFC, BPs accounts for about 70% of the weight, nearly all the volume, and more than 30% of the production cost [1]. Consequently, BPs play essential roles in PEMFC stack assemblies, such as supporting membrane electrode assemblies, enabling gas conductivity, facilitating current collection, and promoting water drainage [2]. Bipolar plates must have corrosion resistance, high conductivity, low cost, strength, and compactness [3]. Currently, hydrogen fuel cell BP materials are commonly classified into three types: graphite plates, composite plates, and metal plates [4]. Graphite sheets are more complex, but their low mechanical strength, restricted electrical conductivity, and expensive processing and manufacturing costs make them unsuitable for general usage in PEMFCs [5]. Composites take a long time to prepare. Thin metallic foils offer high electrical and thermal conductivity, significantly reducing electrochemical stack size. Their adaptable fabrication methods and strong mechanical properties aid in producing ultra-thin BPs [6]. Stainless steel is considered the ideal choice for fuel cell bipolar plates owing to its low cost and superior overall performance compared to precious metals like gold and platinum. Although a passivation layer forms on stainless steel surfaces, PEMFCs operate in an acidic environment. In acidic and electrolytic environments, metal BPs exhibit poor corrosion resistance and surface conductivity [7,8]. Metal ions emitted during corrosion can weaken the electrolyte membrane and harm the electrode catalyst, hindering the proton exchange process, reducing the efficiency of the redox reaction, and ultimately lowering the PEMFC stack performance [9]. Additionally, corrosion forms an oxide layer on the stainless steel surface, interfacial contact resistance (ICR) and limiting the effectiveness of PEMFC stacking. Therefore, to enhance the stainless steel corrosion resistance and electrical conductivity in PEMFCs, the surface of stainless steel bipolar plates must be modified before they can be commercialized. Metal BP coatings for PEMFCs are often modified using vapor deposition processes [10,11,12]. Wang et al. [13] employed a physical vapor deposition approach to manufacture a Pt layer; their experimental findings demonstrate that the Pt layer may efficiently reduce battery resistance, with the best overall performance at 300 W. At 1.4 MPa, the layer reduces from 28.7 mΩ-cm^2^ to 3.54–4.12 mΩ-cm^2^. The ICR change after corrosion is minimal, ensuring good protection for the bipolar plate. Li et al. [14] increased the 334.53 S/cm electrical conductivity and 50.24 MPa flexural strength of PEMFC composite bipolar plates by forming a uniformly distributed multi-walled carbon nanotube network on expanded graphite via in situ chemical vapor deposition. Rashtchi et al. [15] combined plasma nitrogen doping and physical vapor deposition for the first time to apply nanoscale Cr/CrN multilayer coatings on SS 316 L, significantly improving durability through the plasma nitriding layer filling of vapor deposition defects via nitrogen diffusion, with the CrN top layer acting as a chemical barrier. Although the produced coatings have high corrosion resistance and electrical conductivity, traditional methods are prone to more serious interfacial difficulties, whereas the ion implantation process offers many additional areas that can successfully overcome these problems. Mo_2_C stands out among transition metal carbides for its high melting point, thermal stability, and hardness, making it ideal for corrosion-resistant materials in hydrogen fuel cells, especially PEMFC [16]. Feng et al. [17] studied the influence of carbon ion injections on the gradient organization, phase transition, and corrosion resistance of SS 316 L. Carbon ion injection increased the corrosion resistance of SS 316 L by forming an amorphous layer and graphitic solid carbon. Pan et al. [18] studied how Mo inclusion affected the corrosion behavior of (CoNiV)1001-xMox dielectric entropy alloys (MEAs) in 0.5 M H_2_SO_4_ solution. The results suggest that adding Mo elements by microalloying can greatly increase the corrosion resistance of CoNiV MEAs. Zhou et al. [19] investigated the oxidation behavior of a newly developed titanium alloy under 700 °C for 96 h, demonstrating that the L-PBF sample displayed enhanced oxidation resistance compared to the cast metal. This improvement was attributed to the plate-like, large-grained structures, which facilitated optimized pathways for ionic diffusion. Kiniger et al. [20] investigated the sputtering deposition of an Mo (a 100 nm molybdenum) interfacial layer in C/Cu substrates. Following annealing at 600 °C, the Mo layer showed a strong carbon signature, and phase analysis confirmed the synthesis of molybdenum carbide (Mo_2_C). Kim et al. [21] investigated heat and chemical treatments on SS 446 M, confirming their synergy in enhancing corrosion resistance and lowering ICR. Lv et al. [22] investigated ball-milled 2205 DSS with Mo to enhance corrosion resistance in PEMFC cathodes, reducing ICR from 7.5 to 5.2 mΩ-cm^2^ at 500 N/cm^2^. James et al. [23] replaced costly Ti-based BPs with cheaper carbon-based ones. Although untreated carbon plates had lower corrosion resistance in acidic tests, they exhibited stability comparable to titanium plates in real-world conditions. Replacing titanium-based BPs with carbon-based BPs can lead to significant cost savings. Researchers and academics have investigated the safety qualities of stainless steel using thermal processing techniques or coating and heat treatment. This paper investigates the safety qualities of SS 316 L through ion implantation and heat treatment. To enhance conductivity and corrosion resistance in SS BPs, a synergistic strategy of thermal processing and molybdenum–carbon (Mo/C) co-implantation was adopted. Typically, heat treatment of stainless steel alters its characteristics by modifying the microstructure of the substrate [24,25]. This study aims to assess the heat treatment effects on the corrosion resistance and conductivity of Mo-C dual-ion implanted SS 316 L in a PEMFC anode using XRD, SEM-EDS, and AFM.

## 2. Experimental Section

### 2.1. Sample Preparation

The SS 316 L samples exhibited limited-dimensions cylindrical discs (Ø15 mm × 6 mm) cut from SS 316 L sheets. This was undertaken to prevent surface roughness from affecting the samples’ electrical conductivity and corrosion resistance. Before ion implantation, the SS 316 L samples were progressively ground and polished using SiC sandpapers (No. 2000, 5000, 7000, and 10,000 grits) and W1.0 and W0.5 diamond abrasive pastes. The SS 316 L samples were then ultrasonically cleaned in acetone for 20 min at room temperature and subsequently stored in vacuum-sealed containers to prevent oxidation. A Model 50 MEVVA metal vapor vacuum arc ion implanter was used to implant ions into the ground and polished SS 316 L samples. Molybdenum and carbon ions were implanted with a dose of 4.5 × 10^17^ ions/cm^2^ and 6 × 10^17^ ions/cm^2^ at acceleration energies of 40 kV and 30 kV. The ion-implanted samples were heat-treated in a compact tube furnace (OTF-1200X-S, Kejia Furnace, Zhengzhou, China) under Ar_2_ atmosphere conditions, with isothermal conditions holding for one hour at intervals of 200 °C from 600 °C to 1000 °C. The sample codes corresponding to various modification conditions are provided in Table 1.

### 2.2. Surface Characterization

Atomic force microscopy (AFM) (Nanoscience, Phoenix, AZ, USA) analyzed the surface morphology of unmodified and modified SS 316 L samples. SEM-EDS (RTI Laboratories, Livonia, MI, USA) (30 kV, SE detector) identified the elemental distribution and composition of the surface samples. A grazing incidence X-ray diffraction (GIXRD) with an incidence angle of 1° and a Cu target was used for scanning to analyze the physical phase information of the material surface.

### 2.3. ICR Measurements

In PEMFC stacks, the ICR between the BPs and the carbon paper must be evaluated. The ICR of untreated and surface-modified specimens was measured using the sandwich technique to evaluate the effects of ion implantation and thermal processing [26,27,28]. The test setup includes two copper plates on the outside serving as clamping plates and wires attached to the copper plates for electrical conduction. The sample is placed between two layers of carbon paper (Torry TGP-H-900, Toray, Kyodo, Japan) and two parallel copper plates. A steady 0.1 A current flows between the two parallel copper plates, generating a voltage difference from the contact and internal resistances. A pressure of 60–300 N is applied at each side of the device, with voltage readings recorded at various levels.

### 2.4. Electrochemical Measurements

Many researchers and scholars have investigated the performance of SS BPs in simulated PEMFC working settings [29,30,31,32,33,34]. PEMFC operates in a mildly acidic environment (pH 3–6), with solutions containing F^−^, Cl^−^, and SO_4_^2−^, and with temperatures ranging from 60 °C to 80 °C [35,36,37]. To reduce experimental time and expedite the screening of suitable materials, a highly acidic solution, termed an accelerated corrosion solution [38], is typically used for the corrosion resistance testing of BPs. In this study, the corrosion test solution consisted of 0.5 mol/L H_2_SO_4_ and 2 ppm F^−^ [39,40,41,42,43], with the working temperature set at 80 °C. To simulate conditions in actual operation, heating was performed by stirring in an oil bath. Hydrogen was passed through the anode at 20 mL/min. Electrochemical tests were performed on a Shanghai Chenhua 660E workstation (YIMA, Shanghai, China). The test system employed a standard three-electrode configuration. The prepared sample acted as the working electrode, the platinum sheet as the counter electrode, and the saturated mercury sulfate electrode (MSE) as the reference electrode. In advance of electrochemical testing, the samples were ultrasonically cleaned in acetone for 20 min, dried, and mounted in a customized polytetrafluoroethylene fixture with a 1 cm^2^ test area. Before electrochemical testing, the anodic potential was set at −0.5 V (MSE), and the samples were kept at open circuit potential (OCP) until a steady state potential was achieved. Afterward, a dynamic potential polarization test was carried out at 80 °C. To further evaluate the long-term stability of the coatings, constant potential polarization tests were performed, and the ion concentration in the accelerated corrosion solution was examined by the ICP-OES machine.

## 3. Results and Discussion

### 3.1. Characterization of the Modified Layer

The results acquired through various characterization approaches are as follows.

**Figure 1:**
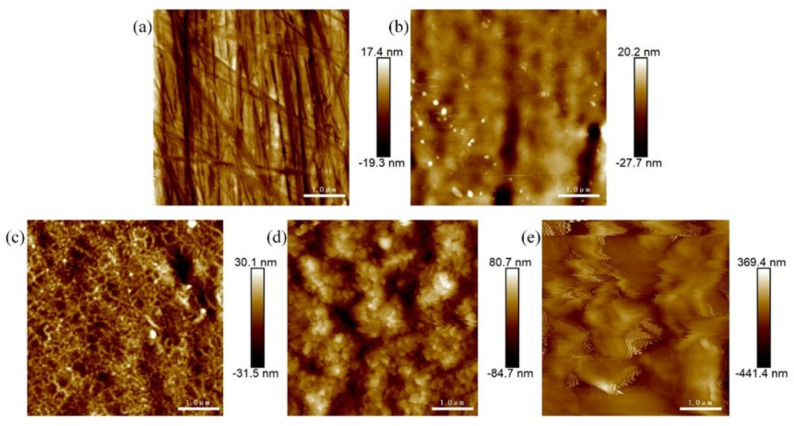
AFM images of SS 316 L under different modifications: (**a**) 316 L; (**b**) (Mo+C); (**c**) (Mo+C)_600-1_; (**d**) (Mo+C)_800-1_; (**e**) (Mo+C)_1000-1_.

**Figure 2:**
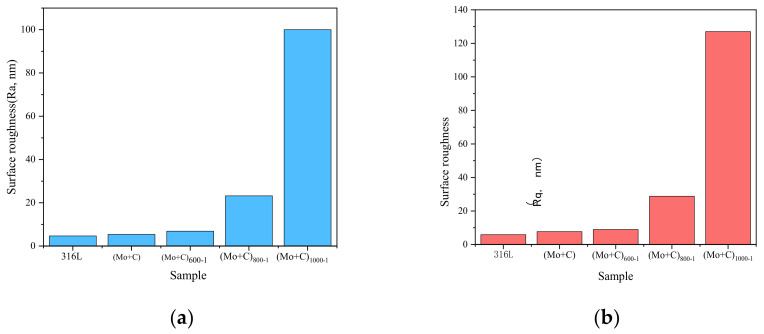
Surface roughness of SS 316 L under different modifications: (**a**) image Ra; (**b**) image Rq.

The AFM (2D) surface morphology of SS 316 L before and after Mo+C dual-ion implantation and heat treatment is shown in Figure 1. As shown in Figure 1a, the surface morphology of 316 L stainless steel without Mo and C dual-ion implantation is generally smooth but exhibits visible scratches. Figure 1b shows the surface morphology of SS 316 L after Mo and C dual-ion implantation, displaying visible surface damage. The original scratches and grooves on the surface of SS 316 L are intensified due to the impulsive and sputtering effects of Mo and C ions. The sputtering effects of Mo and C ions, as shown in Figure 1b, also influence the surface morphology of stainless steel. As the heat treatment temperature increases, the sample surface becomes more uneven, with taller surface spikes, deeper and wider grooves, and increased surface roughness. The (Mo+C)_1000-1_ sample exhibits the most pronounced changes. Figure 2 illustrates the surface roughness of SS 316 L samples under various modifications. The findings show that the surface damage of ion-implanted samples is greater compared to unmodified SS 316 L. This is attributed to sputtering and ion implantation impacts, which enlarge the substrate’s original grooves and gaps. Heat treatment further increased the surface roughness of the samples. The surface roughness of samples heat-treated at 1000 °C for 1 h was approximately 21 times greater than that of unmodified 316 L stainless steel. The above situation is brought about because the heat treatment causes the original and injected components within the stainless steel to react or interact with each other to generate new compounds. It also causes precipitation to the surface of substances such as metal carbides and oxides [44]. These findings demonstrate that Mo and C dual-ion implantation significantly affects the surface morphology of SS 316 L. The thermal treatment temperature of SS 316 L significantly impacts its surface morphology. With increasing heat treatment temperatures, more and thicker oxides precipitate on the surface, leading to greater surface roughness.

**Figure 3:**
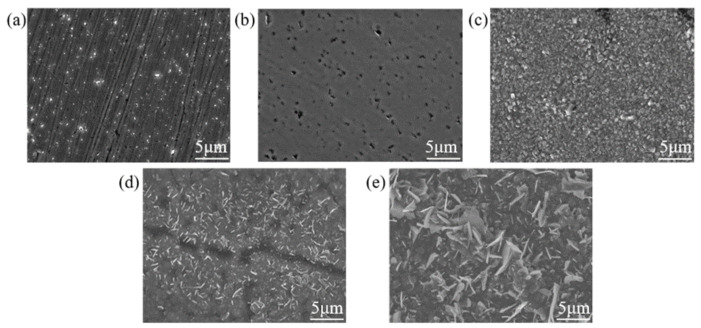
SEM of SS 316 L with different modifications: (**a**) 316 L; (**b**) (Mo+C); (**c**) (Mo+C)_600-1_; (**d**) (Mo+C)_800-1_; (**e**) (Mo+C)_1000-1_.

Figure 3 shows SEM images of SS 316 L samples under different modification conditions. As shown in Figure 3, numerous holes formed on the sample surface after Mo and C dual-ion implantation; these were caused by irradiation damage from high-energy ion bombardment and sputtering during the ion implantation process. The formation of holes increases the contact area between the sample and the corrosive solution, making the surface more prone to localized corrosion and reducing the sample’s corrosion resistance. After heat treatment, significant build-up forms on the sample surface, and its morphology changes considerably with increasing temperature. This is because, under high-temperature heat treatment, the alloy forms a more protective oxide skin during oxidation, which slightly enhances the oxidation resistance of the alloy [44]. This observation aligns with the changes in the surface roughness of the samples shown in Figure 1.

The composition and elemental content of the micro-regions on the SS 316 L surface under each modification parameter are presented in Table 2. Ion implantation significantly increased the Mo and particularly the C content in the samples, which corresponds to the injection dose and confirms the successful incorporation of Mo and C into the SS 316 L substrate.

**Figure 4:**
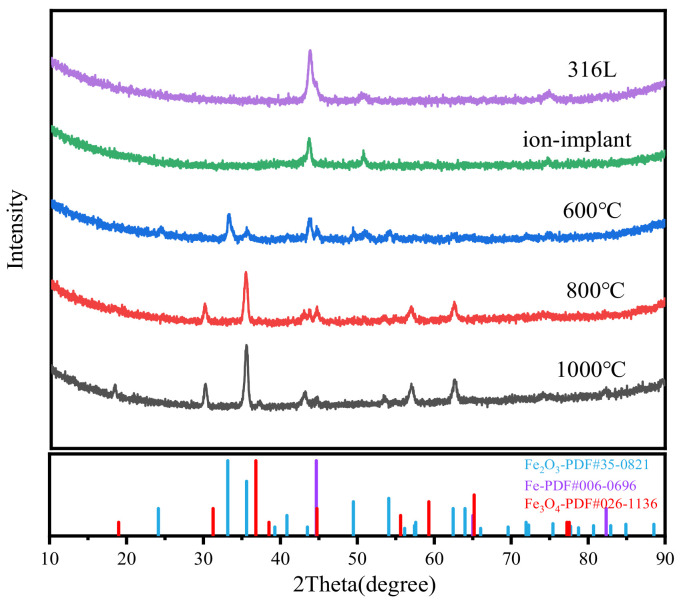
GIXRD plots of SS 316 L under different modifications.

The materials of SS 316 L were analyzed by GIXRD. Figure 4 presents the GIXRD diffraction patterns of SS 316 L, ion-implanted samples, and samples subjected to heat treatment. The surface of virgin SS 316 L is essentially free of oxides, and its diffraction peaks are consistent with those of standard monolithic Fe. The figure clearly shows that when ion injection occurs, the physical phase of the sample and the physical phase of the original 316 L remain consistent, but the diffraction peak broadening, grain refinement, and crystallinity decrease. This is due to the bombardment of high-energy particles in the process of ion implantation, which causes the rearrangement of atoms on the surface of the sample, and defects increase. After heat treatment at 600 °C, new α-Fe_2_O_3_ phases appeared on the sample surface. This is due to the gradual aggregation and precipitation of internal elements toward the interface during heat treatment, which then interact with other metals to generate metal carbides and metal oxides. As the heat treatment temperature rises, the oxide transforms from α-Fe_2_O_3_ to Fe_3_O_4_. This is due to the unstable structure of the oxide layer, which causes oxygen molecules to be desorbed from the surface. As a result, some of the Fe^3+^ is reduced to Fe^2+^, decreasing the degree of oxidation.

### 3.2. Open Circuit Potential (OCP) Measurements and Corrosion Tendency Analyses

Before conducting electrochemical testing, the OCP of the SS 316 L sample must be monitored to ensure the electrochemical stability of the surface. This experiment simulates the PEMFC working environment, where the accelerated corrosion solution is highly acidic, the heating temperature is elevated, the open-circuit test time is prolonged, or the SS 316 L surface is immersed in this environment for an extended duration, potentially impacting the results of subsequent electrochemical tests.

**Figure 5:**
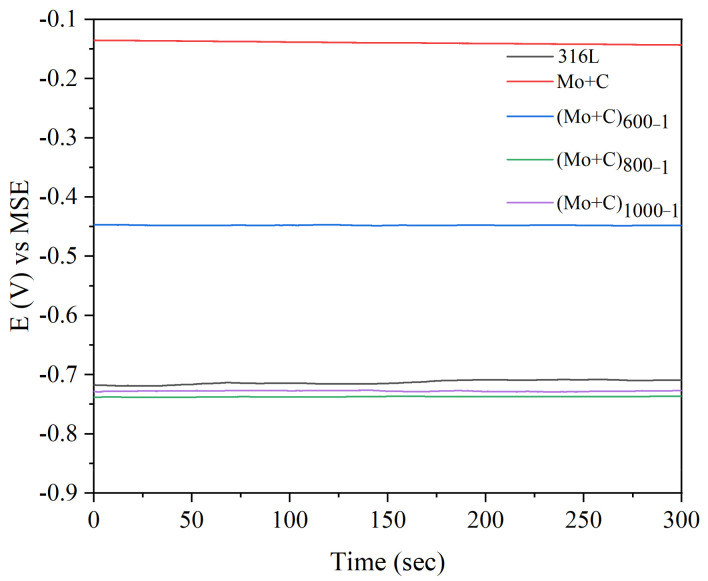
OCP curves of SS 316 L substrates and ion implantation heat-treated samples in simulated anode circumstances.

Figure 5 shows the OCP curves at the anode of the samples before and after the dual-ion injection of C and Mo heat treatment in a simulated accelerated corrosion environment. After C and Mo dual-ion implantation, the corrosion potential on the samples’ surfaces increases significantly. The corrosion potential −0.14 V [MSE] of Mo and C dual-ion-implanted SS 316 L is significantly greater than that of unmodified SS 316 L. In the anodic environment, untreated SS 316 L exhibits a corrosion potential of −0.71 V [MSE]. This occurs because hydrogen in the anodic environment can erode the passivation coating, rendering it unstable and leading to its breakdown and disintegration. The resistance to accelerated corrosion in the anodic environment of C and Mo dual-ion-implanted SS 316 L was significantly improved. The above demonstrates that the presence of Mo and C in the modified layer on the samples surface effectively reduces the corrosion propensity of SS 316 L in an accelerated corrosion environment. The (Mo+C)_600-1_ sample exhibits the highest OCP among the heat-treated samples in the anodic accelerated corrosion environment, with a value of −0.45 V [MSE]. This potential is significantly lower than the anodic OCP of −0.14 V [MSE] observed in the unheated samples, suggesting that heat treatment alters the surface microstructure and chemical composition, enhancing corrosion resistance.

### 3.3. Dynamic Potentiodynamic Polarization Scanning and Corrosion Rate Analysis

**Figure 6:**
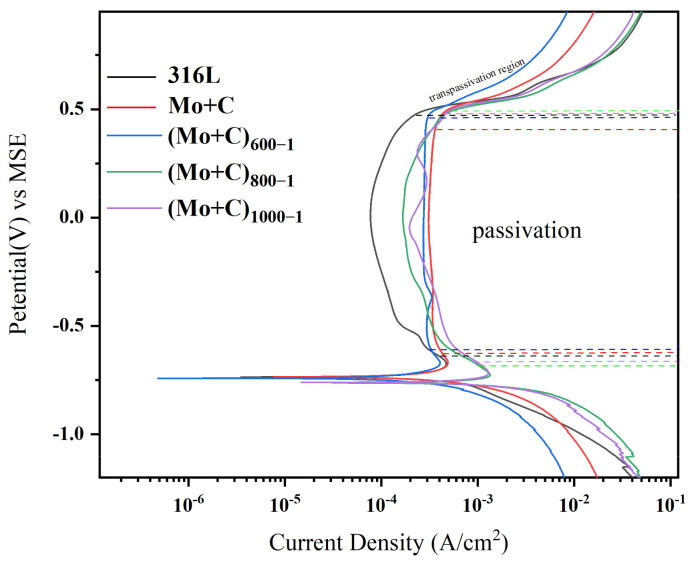
Anode polarization curves of SS 316 L substrates and heat-treated samples with ion implantation under simulated anodic conditions.

Dynamic potentiodynamic profiling and corrosion rate analysis were conducted on stainless steel substrates and ion-implanted heat-treated modified samples, yielding the following results. Figure 6 depicts the curves about the potentiodynamic polarization for each sample in the anode environment. Table 3 displays the corrosion data derived from the fitting of potentiodynamic polarization curves.

Table 3 shows the corrosion current densities (I_corr_) and corrosion potential (E_corr_) for various samples, with the (Mo+C)_600-1_ sample having the lowest value (5.32 × 10^−4^). Its I_corr_ is significantly lower than that of the SS 316 L sample, attributed to the formation of surface metal carbides (Mo+C). The (Mo+C)_600-1_ sample exhibits better surface corrosion resistance. The I_corr_ of the ion-implanted sample is comparable to that of the unmodified SS 316 L sample. This similarity may result from surface defects and irradiation damage from ion implantation, which is consistent with the AFM and SEM data. The passivation film generated on the surface was unstable, resulting in corrosion degradation.

The dynamic polarization curves of the samples pre- and post-heat treatment with Mo and C double-ion implantation in an anodic accelerated corrosion environment are shown in Figure 6. As shown in Figure 6, the corrosion potentials of the samples range between −0.6 and −0.8 V. The passivation current density of the ion-implanted sample (approximately 3.08 × 10^−4^ A/cm^2^) is significantly higher than that of the unmodified SS 316 L samples. However, the corrosion resistance of pristine C and Mo dual-ion-implanted SS 316 L samples in an anodic accelerated corrosion environment was lower than that of unmodified SS 316 L samples. The fundamental cause for this predicament is that during the C and Mo ion implantation process, the high-energy ion beam bombardment of the SS 316 L surface creates a huge number of holes, causing irradiation damage to the SS 316 L surface. Hydrogen in the anodic environment is more likely to penetrate the pores on the surface of the sample, making the passivation film on the ion-implanted sample surface unstable and prone to local corrosion. The heat-treated samples all exhibited self-passivation in the accelerated corrosion solution, displaying a characteristic corrosion profile for SS 316 L, including a cathodic polarization zone, an anodic dissolution activation zone, a transition zone, a passivation interval, and an overpassivation zone [24,25]. The self-corrosion current was reduced by approximately 50% following heat treatment, showing that the corrosion resistance was much better than that of the non-heat-treated samples. The kinetic potential polarization curves of the heat-treated modified samples were more similar in the anodic accelerated corrosion environment. In the anodic accelerated corrosion environment, the corrosion potentials of the (Mo+C)_1000-1_ and (Mo+C)_800−1_ samples were approximately −0.76 V. At 600 °C, the V_corr_ of the (Mo+C)_600-1_ sample rose to about −0.73 V, while the passivation currents were significantly lower than those of the (Mo+C)_1000-1_ and (Mo+C)_800-1_ samples. Meanwhile, the self-corrosion current density of the samples heated at 600 °C was much lower than that of the (Mo+C)_1000-1_ and (Mo+C)_800-1_ samples, indicating that a specific heat-treatment process can significantly enhance the corrosion resistance of SS 316 L. The I_corr_ of the (Mo+C)_600-1_ sample was much lower than that of the SS 316 L and ion-implanted sample, indicating that the 600 °C heat treatment effectively prevents the corrosion of the SS 316 L BP in corrosive solutions.

### 3.4. Static Potential Current–Time Curve and Corrosion Resistance Stability Analysis

The static potential current–time curve and corrosion resistance stability analysis were used to completely analyze the materials’ corrosion resistance and determine their suitability for acidic application settings.

**Figure 7:**
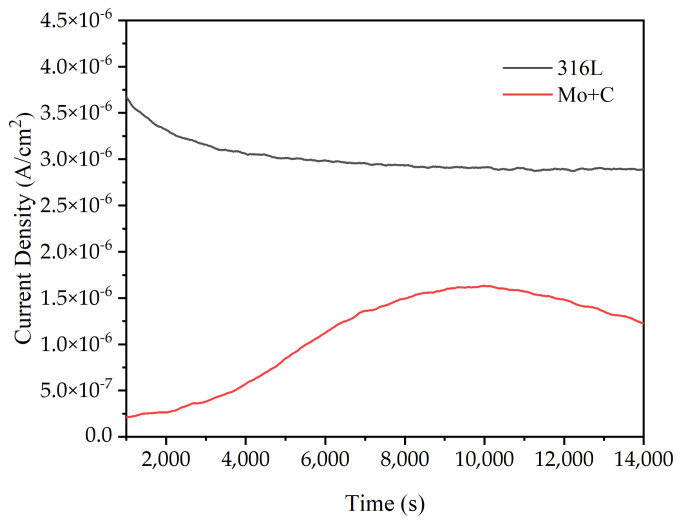
Electrostatic potential current–time curves of SS 316 L substrate and ion-implanted samples in simulated anodic conditions.

The electrostatic potential current–time curves for the stainless steel substrate and the ion-implanted sample in the simulated anode condition are shown in Figure 7. To investigate the stability of bipolar plate materials for long-term operation in a PEMFC environment, the samples were subjected to an electrostatic potential polarization corrosion test at 80 °C, with H_2_ flowing at a rate of 20 mL/min into a 0.5 mol/L H_2_SO_4_ and 2 ppm F^−^ [39,40,41,42,43] accelerated corrosion solution. The electrostatic potential polarization test can disclose the changing laws of I_corr_ on the sample surface over time. Thus, the curve was utilized to assess the stability and dependability of naked and modified 316 L samples in the accelerated corrosion solution over time. The figure shows that the corrosion current densities at the anode of the unaltered SS 316 L sample remained at approximately 3 μA/cm^2^. After Mo and C double-ion injection, the corrosion current density on the sample surface appeared to be substantially lower in the anodic environment than on bare stainless steel, and it fluctuated within a specific range. The corrosion current densities of the ion-implanted samples in the anodic environment increased and then decreased, indicating that the original passivation layer on the sample surfaces was disrupted and a new passivation film formed, which remained stable.

**Figure 8:**
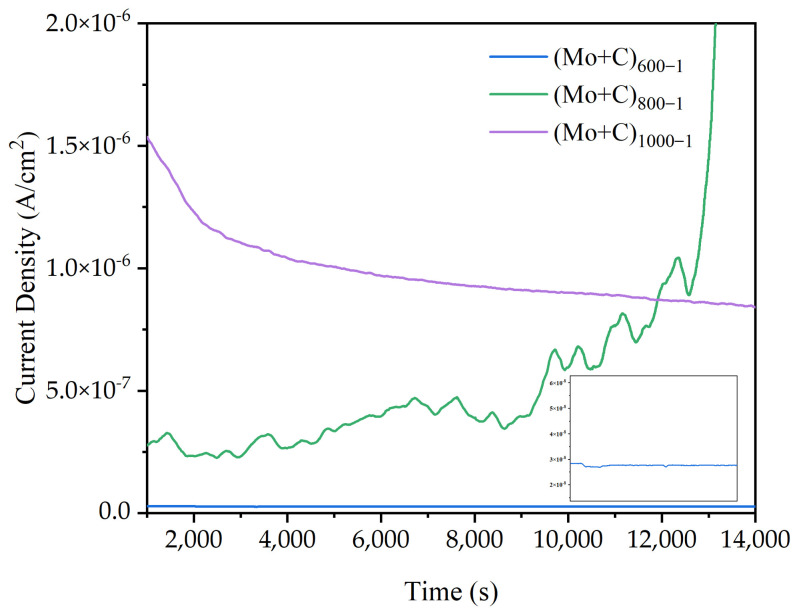
Potentiostatic curves of heat-treated samples in simulated anode conditions.

Each sample’s electrostatic potential current–time curves in a simulated anodic accelerated corrosion environment are displayed in Figure 8. The stability and dependability of the heat-treated modified samples in an accelerated corrosion solution for long-term operation were examined using current–time curves at constant potential. The constant potential value was −0.5 V (MSE) in the simulated anode working environment. Except for the (Mo+C)_800-1_ sample, the corrosion current densities progressively stabilized following four hours of electrostatic potential polarization. This shows that the dissolution and formation of a passivation film in this state have been in dynamic equilibrium, and the relative stability of the passivation film requires a very low corrosion current density, so the corrosion current density reaches a stable state. (Mo+C)_800-1_ samples exhibit apparent fluctuations because the passivation film formed on the sample’s surface is unstable, resulting from the rupture and then the passivation process. As the modified temperature increases, the fluctuation reduces and becomes smoother, such as (Mo+C)_1000-1_. This is because a thicker oxide layer might grow on the material’s surface at 1000 degrees Celsius. Oxide layers have low electrical conductivity, and their thickening can inhibit the movement of charge carriers, lowering current density, with no additional passivation occurring. Meanwhile, the corrosion current density grew gradually as the heat treatment temperature rose. The (Mo+C)_600-1_ sample had a much lower I_corr_ (about 0.26 μA/cm^2^) than other heat-treated samples, as well as that of the ion-implanted sample and the unmodified SS 316 L sample, indicating that heat treatment of stainless steels alters their properties by changing the microstructure and chemical composition of the matrix [45,46]. The results show that carbides and oxides from surface precipitation can prevent corrosion and improve sample corrosion resistance under high-temperature heat treatment at 600 °C for 1 h. The test results of the major metal ion concentrations of each heat-treated sample in the anodic accelerated corrosion environment are given in Table 4. Because of Fe selective solubility, the anode contained the largest concentration of Fe ions. In the anodic environment, the corrosion solution of the (Mo+C)_600-1_ sample had the lowest concentration of metal ions, which was much lower than the other samples. The concentration of metal ions in the (Mo+C)_600-1_ sample solution was 40–80% lower than that in the ion-implanted sample solution and 40–90% lower than that dissolved in the unmodified SS 316 L sample, indicating superior corrosion resistance. The metal ion concentration in the (Mo+C)_600-1_ sample solution was lower than that in the ion-implanted sample solution, owing to the formation and precipitation of metal carbides and metal oxides on the sample surface under 600 °C heat treatment, which partially repaired the irradiation damage caused by ion implantation. The concentration of main metal ions in each heat-treated sample’s corrosion solution matches the results of the kinetic potential polarization test.

### 3.5. Interfacial Contact Resistance After Anodic Corrosion

Interfacial contact resistance is a key measure of battery performance. The ICR measurements under ion-implantation heat treatment conditions on stainless steel substrates are as follows.

**Figure 9:**
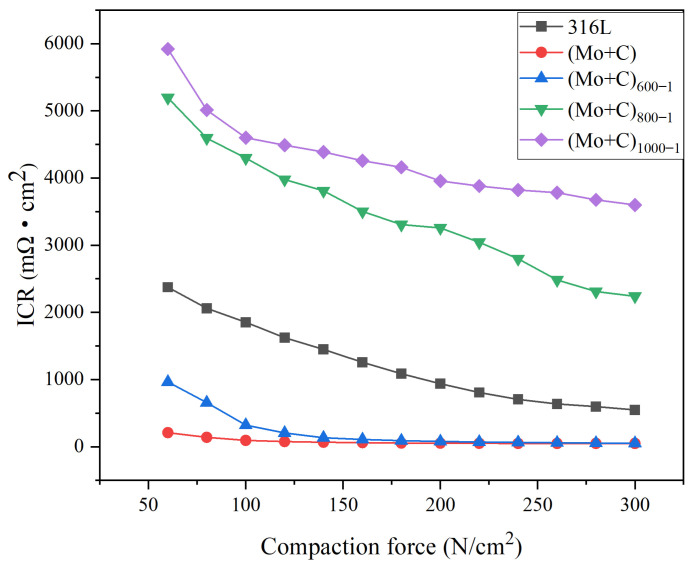
ICR curves of SS 316 L samples under different heat treatment conditions at the anode.

Interfacial contact resistance (ICR) is a key indicator of cell performance. In our previous study, we found that untreated 316 L stainless steel had relatively high surface contact resistance [38], while after Mo and C ion injection, the samples showed significantly lower ICR values compared to untreated 316 L stainless steel. It has been demonstrated that Mo and C ions after double injection have an active and beneficial effect on the bipolar plate’s conductivity. The results of the anode heat treatment ICR measurements are shown in Figure 9. With increasing compaction force, the ICRs of the samples pre- and post-ion injection, in addition to heat-treated samples under various circumstances, follow similar trends. ICR decreased initially in the low-pressure range (60–140 N/cm^2^) and then gradually decreased until it stabilized at compressive forces greater than 200 N/cm^2^. The aforementioned situation occurs mostly because the actual contact area between the carbon paper and the sample is limited at low-pressure strength conditions, and the conductivity is weak, resulting in a high ICR. As the pressure increases, the deformation of the carbon paper increases the number of sample contact points, resulting in more conduction channels and a lower ICR value. At the same time, the surface roughness, shape, and content of the passivation film are significantly influenced by heat treatment on stainless steel bipolar plates. As a result, they affect the bipolar plates’ surface contact and corrosion resistance [46]. Figure 9 shows that the heat-treated samples had a higher ICR after anodic polarization. The conductivity of the passivation layer is reduced during the polarization process because Fe has strong selective corrosion, and the thickness of the passivation layer also contributes to the increase in ICR value. In an anodic environment, the ICR value is positively associated with the heat treatment temperature; that is, the greater the heat treatment temperature, the lower the surface conductivity after polarization. After electrostatic potential polarization at the anode, the (Mo+C)_600-1_ sample had a much lower ICR value than the untreated SS 316 L sample. The decreased ICR under 600 °C heat treatment settings could be due to the metal carbides that form on the surface at this temperature, which increases its electrical conductivity.

## 4. Conclusions

Ion implantation and heat treatment formed a non-homogeneous layer on SS 316 L. In comparison to untreated SS 316 L, Mo and C double-ion implantation causes irradiation damage on the surface due to high-energy sputtering, and the electrical conductivity improves dramatically, but the corrosion resistance does not. During heat treatment, particularly at 600 °C for 1 h, conductive and corrosion-resistant carbides formed on the stainless steel surface, partially repairing ion implantation-induced irradiation damage. The I_corr_ of the 600 °C sample in the kinetic potential test (5.32 × 10^−4^ A/cm^2^) was 54% lower than that of bare SS 316 L (1.17 × 10^−3^ A/cm^2^). In the electrostatic potential test, the corrosion current of the 600 °C sample stabilized at a low value (about 0.26 μA/cm^2^), with the lowest concentration of dissolved metal ions (Fe^2+^ 2.908 mg/L). After anodic electrostatic potential polarization, the ICR of (Mo+C)_600-1_ was found to be much lower than that of untreated SS 316 L. Electrochemical tests show that (Mo+C)_600-1_ samples have better passivation and corrosion resistance than untreated SS 316 L in simulated PEMFC anode conditions. Electrochemical studies confirm the improved passivation and corrosion resistance of (Mo+C)_600-1_ in simulated PEMFC anode conditions. Therefore, (Mo+C)_600-1_ holds significant potential as a BP for PEMFC applications.

## Figures and Tables

**Table 1:** Sample codes corresponding to different modification conditions.

Sample Code	Bare SS316 L	Ion Implantation Mo: 4.5 × 10^17^ ions/cm^2^, 40 kV C: 6 × 10^17^ ions/cm^2^, 30 kV	Heat Treatment Temperature (℃)	Heat Treatment Time (h)
(Mo+C)_1000-1_	-	√	1000	1
(Mo+C)_800-1_	-	√	800	1
(Mo+C)_600-1_	-	√	600	1
(Mo+C)	-	√	-	-
316 L	√	-	-	-

√: use the conditions; -: not use the conditions.

**Table 2:** Elemental mass concentration of SS 316 L samples under different modification conditions.

Sample	Element Mass Concentration (Wt%)			
	Fe	Mo	C	O
(Mo+C)_1000-1_	38.24	0.99	14.71	46.05
(Mo+C)_800-1_	61.08	2.02	13.05	23.86
(Mo+C)_600-1_	55.52	1.67	19.03	23.78
(Mo+C)	67.10	2.08	25.21	5.61
316 L	73.85	2.03	19.53	4.59

**Table 3:** The polarization test results of different samples under anodic simulation conditions.

Sample	E_corr_ (V)	I_corr_ (A/cm^2^)	I_pass_ (A/cm^2^)
(Mo+C)_1000-1_	−0.59	5.36 × 10^−4^	3.21 × 10^−4^
(Mo+C)_800-1_	−0.78	5.93 × 10^−4^	3.02 × 10^−4^
(Mo+C)_600-1_	−0.60	5.32 × 10^−4^	3.38 × 10^−4^
(Mo+C)	−0.80	1.21 × 10^−3^	3.08 × 10^−4^
316 L	−0.80	1.17 × 10^−3^	1.61 × 10^−4^

**Table 4:** Key metal ion concentration in anodic corrosion solutions.

Sample	Ionic Concentration mg/L			
	Fe	Mo	Ni	Cr
(Mo+C)_1000-1_	3.438	0.288	1.845	2.071
(Mo+C)_800-1_	19.482	1.557	2.898	4.579
(Mo+C)_600-1_	2.908	0.062	0.260	0.375
(Mo+C)	4.387	0.312	1.905	2.212
316 L	5.983	0.635	2.130	3.214

## Data Availability

Dataset available on request from the authors.
